# Gait disorders in unipolar and bipolar depression

**DOI:** 10.1016/j.heliyon.2023.e15864

**Published:** 2023-05-01

**Authors:** Diana Bogdanova

**Affiliations:** Department of Psychiatry and Medical Psychology, Medical University of Sofia, 15 Akademik Ivan Evstratiev Geshov Blvd, Sofia, Bulgaria

**Keywords:** Unipolar depression, Bipolar depression, Psychomotor skills gait disorder

## Abstract

**Objectives:**

Bipolar and unipolar depressions have a similar clinical picture, but different neurological and psychological mechanisms. These misleading similarities can lead to overdiagnosis and increased suicide risk. Recent studies show that gait is a sensitive objective marker for distinguishing the type of depression. The present study aims to compare psychomotor reactivity disorders and gait activity in unipolar and bipolar depression.

**Methods:**

A total of 636 people aged 40.7 ± 11.2 years are studied with an ultrasound cranio-corpo-graph. They are divided into three groups - patients with unipolar depression, with bipolar depression and healthy controls. Each person performs three psychomotor tasks - a classic Unterberger task, a simplified version with open eyes and a complex version with an additional cognitive task.

**Results:**

We find significant differences in psychomotor activity and reactivity between the three groups. Bipolar patients have more inhibited psychomotor skills than unipolar and they are both more inhibited than the norms. The simplified variant of the equilibriometric task is the most sensitive one and psychomotor reactivity is a more precise marker than psychomotor activity.

**Conclusion:**

Both psychomotor activity and reactivity in gait could be sensitive markers for distinguishing similar psychiatric conditions. The application of the cranio-corpo-graph and the possible development of similar devices could lead to new diagnostic and therapeutic approaches, including early detection and prediction of the type of depression.

## 1 Introduction

In clinical practice, the mental health norm is determined by three characteristics – relevance, timeliness and commensurability. We can assume that a deflection in any of these three indicators is an indication of a deviation in behavior or adaptive response. The combination between the movements of the facial muscles and the muscles of the body, reflecting the inner mental life of the person, is called *psychomotor skills*. The combination of various qualitative and quantitative disorders of affect, cognition and will, expressed through elements of psychomotor skills (such as gait, posture, speech, facial expressions, etc.) is defined as *psychomotor disorders* (PD).

In depression PDs are divided into two types, according to the direction of the deviation from the norm – agitation and retardation [1]. In practice, we can find that in the case of depression these polar psychomotor disorders are characterized by activation in different neurocerebral structures and neurotransmitter pathways. Modern technologies allow us the visualization of pathognomonic changes in the structures of the brain in various mental disorders. Among depressive patients we can find very mixed data on the volume of the prefrontal cortex [2], changes in the cerebellum [3], Raphe nucleus [4], nigrostriatal pathway [5], hippocampus [6], white matter [7], frontal and prefrontal lobes in the brain [8,9], amygdala [10,11], hypothalamus [12], serotonin and dopamine pathways [13]. The described changes in the volume of the cited brain structures are associated with both the type of actual psychomotor disorder (PD) and the level of neurocerebral plasticity exhibited during the depressive episode (DE). In depression with psychomotor agitation, abnormal changes in serotonergic structures of the prefrontal cortex, mesocortical and mesolimbic dopaminergic pathways in the brain and changes in the amygdala and its adjacent hypothalamic-pituitary-adrenal axis are most often found in the literature. Second, studies reporting frontal lobe dysfunctions and mutations in the catechol-*O*-methyltransferase gene involved in dopamine metabolism and catecholamine activation among psychomotor agitation in patients with agitation in schizophrenia and in bipolar disorder [14].

In this direction, recent studies by Haralanov and team show that agitation is recorded in depressed individuals with striatal *hyperdopaminergic*, while psychomotor retardation in depression is associated with underlying nigrostriatal hypo-dopamine energeia [15]. Polar neurobiological characteristics in the field of psychiatry are associated with diametrically opposed drug therapies [16,17].

The problem is that the phenomenon of agitation and retardation, according to ICD-10 and DSM-5, could be a part of recurrent depressive disorder (RDD) and bipolar affective disorders (BAR). In psychiatry, the activation of opposite psychomotor disorders is associated with various cortical structures and neurotransmitters. This suggests using the opposite drug treatment for individual types of depression [18]. According to many clinicians, a large percentage of BAR cases present as RDD. The risk of mixed depression being diagnosed as pure unipolar depression or RDD is much bigger than RDD being diagnosed as mixed depression [19] or BAR. This leads to wrong diagnosis and wrong medication. As a result, the suicidal risk increases, and this leads to the need to find objective methods for the prevention of depression.

In recent decades, number of non-invasive technologies have been introduced to register psychomotor disturbances in individuals with a vestibular or psychiatric problem. Depressed individuals have been found to have a slower walking speed compared to non-depressed individuals [20]. Some studies have reported impaired stride length, with shorter stride length and less arm swing, as well as reduced vertical head movement, shoulder range of movement, and elbow movement during stepping [21]. Increased body sway compared to non-depressed individuals has also been described [20]. Other studies have shown positive correlations between current symptoms of depression and motor behavior. It is report for increased periodicity in motor activity characterized by a combination of reduced activity and occasional bursts of activation as depressed mood deepens [22,23].

Recent studies with actigraph show that this method is increasingly used in psychiatric practice in combination with other sensors that report changes in different parts of the body – wrist, ankle, waist, etc. [24]. When it is comparing unipolar with bipolar depression, differences in activity between the two types of depressed patients are reported, which are strongly expressed in the time interval 6.00–12.00 h. According to the same study, with the same severity of depressive symptoms, bipolar patients are more psychomotor retarded than unipolar individuals [25]. Another objective study also shows that individuals with unipolar depression are more psychomotor activated than individuals with bipolar depression [26]. Many other studies have shown that the most sensitive marker in the study of PD in depressed patients is gait [27]. The present work focuses on the objective study of PD manifested in gait in endogenous unipolar and bipolar depression.

When we use the term *unipolar depression*, according to the ICD-10 criteria, we are referring to that mental disorder known under clinical code F33, such as recurrent depressive disorder (RDD). According to the diagnostic criteria of the same manual, we assume that in order to be diagnosed with RDD by a psychiatrist, there must be evidence of cyclic depressive states, called depressive episodes. Between depressive episodes a short or long-lasting stage of enlightenment (intermissions) may appear, which is expressed in a return to the typical functioning of the personality, without evidence of an elevated mood satisfying the criteria for mania.

When we talk about *bipolar depression*, we are referring to the psychiatric suffering known in the same manual under clinical code F31, as bipolar affective disorder (BAR). According to the diagnostic criteria of the same manual, we assume that in order to be diagnosed with BAR by a psychiatrist, there must be evidence of repeated (at least two) episodes in which the patient's mood and activity are significantly disturbed in the patient's history. These mood disorders, on the one hand, should be expressed in an increase in energy and activity (up to a manic or hypomanic episode), and on the other – in a decrease in energy and activity (a depressive episode). The main thesis of the present article is that statistically significant differences can be found between the two similar depressive states. The aim of our study is to compare two gait parameters – number of steps per minute (psychomotor activity) and lateral sway (psychomotor reactivity) in patients with unipolar and bipolar depression in a moderately severe depressive episode.

## 2 Methods

The design of the study is between-group – each of the participants from the three groups (RDD, BAR and Healthy controls) went through all the psychomotor tasks individually, after visually and verbally given instructions by the researcher. All subjects are individually examined by cranio-corpo-graphy (CCG). This is a wireless and non-invasive computerized method developed by Clausen in 1968. The CCG method was created in order to study the equilibrium functions in norm and in pathology in occupational medicine. The study of these functions is accomplished with the help of a specially designed computer program called “Win Balance”. The program detects the changes in the length of the ultrasonic waves emitted from the CCG body to the sensors attached to the participant and records in visual and digital form the parameters of the initial and final position of the object and its changes in its movements. Sound is moving through air with an average velocity of 330 m per sec. By means of 3 microphones in fixed arrangement in space the sound signal can be precisely located according to its source. This is based on a mathematical analysis. This principle is installed into the ZEBRIS Coordinate Measurement System® [28]. This allows objective and accurate visualization of movements, as well as quantitative recording of the parameters of movement of the head and body in patients. By using the CCG method we can explore five psychomotor parameters, while participants are performing various equilibriometric tasks. These five parameters are:

1. Long deviation – unconscious movement in space from the initial to the final position of the study, measured in centimeters
2. Lateral sway – unconscious medial-lateral swaying during each individual step as a result of the automated anteropulsion during human gait (the maximum-minimum value is taken into account), measured in centimeters
3. Angle deviation – angular deviation from the initial to the final position of the examined person, measured in degrees
4. Self spin – unconscious rotation around the rest of the body, reflecting asymmetry in locomotion, measured in degrees
5. Steps – number of steps per minute (cadence)

These CCG parameters are registered and archived automatically in the database of the program Win Balance. Their values are seen in digital form with their adjacent measurement units, and they are accompanied by a graphic visualization of the movements in space. The resulting visualization of the movements is called a cranio-corpo-gram [29]. Each marker is displayed in a different color on the monitor to easily stand out from the rest. Through the data obtained, we can identify recurring patterns in the individual psychiatric and non-psychiatric groups. Normative values for each parameter are adapt to a clinically healthy European sample performing the classic Unterberger-Fukuda and Romberg task. The normative values of individual motor parameters are indicated on the cranio-corpo-gram in rectangles colored in light green, and pathology – in dark green and red. The normal values in the stepping test of the lateral sway are between 5.17 cm and 16.15 cm. The mean value of the angular deviation is 55.13° to the right and 48.37° to the left. The body spin mean value is 82.21° to the right and 82.89° to the left [28]. Normative values for the number of steps taken per minute in clinically healthy participants is between 80 and 100 [29]. The automated generation of the results allows easy, fast and objective identification of possible pathologies in each participant, without the need for special training for the work of the researcher. This method is not expensive. It is affordable and easy to replicate in laboratory conditions. CCG is an important achievement in the toolbox of equilibriometry for regular and special neurootological investigations. It can be used effectively in monitoring the effect of therapy in psychiatric patients and their subgrouping according to the type of their psychomotor disorder.

In psychiatric practice, the CCG method was introduced by Clausen, Haralanov and co-workers in 1994, and this method and its similar modifications are still used today. In the process of work, over the last three decades, a number of subtle and significant differences in psychomotor reactivity and activity in similar psychiatric disorders have been identified, requiring different therapeutic interventions [30]. In previous studies on psychiatric patients, cranio-corpo-graphic pathology was registered – with abnormal movement patterns in the direction of increased forward movement or reduced forward and backward movement when patients performing the Unterberger-Fukuda task. The presence of dysrhythmic lateral sway of the body, changes in the direction of movement, significant differences in the number of steps the patient takes in 1 min are also reported [31].

In achieving the set goal through research with the CCG method, three psychomotor tasks are applied with increasing difficulty. One of them is the classic equilibriometric task of Unterberger-Fukuda. It is performed by the subject with closed eyes (CE), while the participant from the starting position upright, steps in the same place with knees bent below 90° and arms and fingers outstretched. The other tasks modify the classical method of Unterberger on the idea of Haralanov. The simplified version of the task is performed by the subject with open eyes (OE). The complicated version includes a cognitive task – the subjects have to count down from 100 with their eyes closed. The three equilibriometric tasks are dynamic in nature and are performed individually by the subjects within 1 min in a well-lit and soundproof laboratory. After 60 s, the computer releases a sound signal, indicating that the patient shall stop. Then the test results are analyzed graphically and numerically. The movement pattern is graphically adjusted to the center of a polar coordinate system, from where the test started. This means that the starting position of the test person is not bound to a concise starting point. The adjustment is performed by the program [29]. The average time for examining one person is 6 min. Before each psychomotor task, the researcher explains and shows the next task to be performed. This means that between different tasks the participants do not get physically tired. Their purpose is to observe and listen to the researcher what his next task is.

### 2.1 Participants

The study was approved by the local Ethics Committee of the First Psychiatric Clinic at the University Multidisciplinary Hospital for Active Treatment in Neurology and Psychiatry (MHATNP) “St. Naum” – Sofia. It was held in the period December 2017–March 2020, on the territory of the First Psychiatric Clinic, in the “Laboratory of Neurophysiology” at MHATNP “St. Naum”, Sofia, Bulgaria.

*Inclusive criteria:* The study included all psychiatric outpatient and non-outpatient patients aged between 19 and 60 years, treated at MHATNP “St. Naum” and diagnosed by psychiatrist with either F.33 RDD or F.31 BAR in a moderately severe depressive episode, without other psychiatric, neurological and/or organic disorders that could affect psychomotor skills. All patients with depression are examined with CCG before starting their drug therapy. The diagnoses were based on the detailed case history study and the clinical findings of the Unterberger-Fukuda blind walking task. In the study, we also include all clinically healthy individuals aged between 19 and 60 years who have no evidence of cranio-corpo-graphic pathology or other diseases (neurological, psychiatric or organic). All participants sign an informed consent to participate.

*Exclusion criteria:* All participants who come to the clinic with prescribed and accepted drug therapy are excluded from the study. Participants with a history of traumatic brain injury, organic diseases, accompanying autoimmune, neurological and/or other psychiatric diseases were also excluded from the study. Any incomplete or interrupted cranio-corpo-graphic recordings are voided from the study. Results where the participant withdraws their consent to participate are also disqualified.

## 3 Results

### 3.1 Descriptive statistics and statistically significant differences

During the primary processing of the CCG records from all 1068 examined participants, it was necessary to cancel the data of 432 subjects (Ss). The final working sample in the present study reached a total of 636 people. They are divided into three groups. The first group consists of 317 people diagnosed with recurrent depressive disorder (182 women and 135 men), aged 43.4 ± 11.9. The second group – 137 people diagnosed with bipolar disorder (70 women; 67 men), aged 41.3 ± 11.1. The third one includes 182 people – healthy controls (HC), of whom 98 were women and 84 were men, aged 38.0 ± 9.7. The demographic data of the subjects (Ss) in the methods used are presented in Table 1.

**Table 1:** Demographic data of subjects with CCG.

	RDD	BAR	NORMS
**Total number Ss**	N_(RDD)_ = 317	N_(BAR)_ = 137	N_(Norms)_ = 182
Sex	N_(F)_ = 182; N_(M)_ = 135	N_(F)_ = 70; N_(М)_ = 67	N_(F)_ = 98; N _(M)_ = 84
Age	43.4 ± 11.9	41.3 ± 11.1	38.0 ± 9.7
Weight	73.0 ± 15.1	76.1 ± 15.2	71.7 ± 14.9
Height	168.8 ± 11.3	172.0 ± 8.7	171.8 ± 10.2

Table 1 shows that the number of samples is unevenly distributed. The largest share of the total sample is occupied by persons with RDD at 49.8%. The second largest is the number of clinically healthy people – 28.6% and the third – diagnosed with BAR – by 21.5%. The three working samples – RDD, BAR and NORMS, are dominated by women.

The SPSS program – version 19 for Windows was used for statistical processing of the obtained data. For the CCG parameters “Lateral sway” and “Steps” in the OE, CE and COCE task, we make box-plot diagrams, in order to find and remove deviations (outliers) and extreme outliers. In the case of an extreme value, a correction is made, and the nearest non-extreme value is written in its place. We should note that some of the studied persons have already been excluded, due to valid reasons described in the “exclusion criteria” section. As a second step of the primary processing of the results, a test for normality of the data (Kolmogorov-Smirnof and Shapiro-Wilk) is performed. The result of the tests showed that the data does not have a normal distribution and should be normalized. In such cases, a two-step approach should be used for this purpose [32], namely:

- Creating an auxiliary variable based on grading the results (fractional rank cases);
- Application of an inverse distribution function on the scaling variable (Inverse Distribution Function – IDF. Normal).

As a result, a normalized value is obtained for each of the CCG indicators “Lateral sway” and “Steps” in the three psychomotor tasks – OE, CE, COCE. After checking for outliers in the three samples and removing them, we calculate descriptive statistics for the three groups on the three psychomotor tasks. Data are presented as mean values with standard deviation (SD). Statistical analysis was performed using descriptive statistics, parametric and non-parametric analyses (analysis of variance ANOVA, cross-tabulation, factor analysis, post hoc Scheffe, Games-Howell, Levene's test) using SPSS v.19 IBM Software. Differences were considered statistically significant at p < 0.05. Cross-tabulation descriptive statistical analysis was applied for the identification of the manifested psychomotor reactivity (PMR) and psychomotor activity (PMA) in gait in three groups (RDD, BAR and NORMS). When we are recording the levels of psychomotor reactivity (PMR), the results of the CCG variable “Lateral sway” are taken into account, while when we are examining psychomotor activity (PMA) the values obtained from the variable “Steps” are calculated. According to previous studies, it is assumed that the high values of CCG parameter “Lateral sway”, above the average value of the norm for healthy controls, indicates the presence of brady-reactivity. Decreased values on the same indicator indicate tachy-reactivity. Increased values of CCG indicator “Steps” indicates the presence of hyper-activity, and decreased values of the same variable-hypo-activity. The combination of brady-reactivity and hypo-activity in the same depressed person is indicative of inhibited psychomotor skills, while the available tachy-reactivity and hyper-activity is interpreted as an indicator of activated psychomotor skills [24,25]. Pearson's cross-tabular analysis with the post-hoc analysis of adjusted residuals shows that in terms of reactivity there is a significant difference in the percentage distribution between the two clinical groups presented in Table 2.

**Table 2:** Percentage distribution of tachy- and brady-reactive subgroups.

Groups	Tachy-reactive		Brady-reactive	
Task	RDD	BAR	RDD	BAR
**OE**	26.2	16.8	73.8	83.2
**CE**	27.8	16.8	72.2	83.2
**COCE**	26.2	31.4	73.8	68.6

In Table 2, we see that in the OE task 26.2% of patients with RDD have manifest tachy-reactivity in gait. This means that nearly a quarter of the examined patients with RDD take a lower lateral sway than the average of the norm. In contrast tachy-reactivity in gait in BAR patients occurred in 16.8% of participants. On the other hand, in the same task brady-activity in the gait in RDD was manifested in 73.8% of the participants, while this percentage in BAR was 83.2%. In the CE task 27.8% of patients with RDD have manifest tachy-reactivity in gait and 16.8% of BAR participants showed tachy-reactivity. This means that a larger population of participants with RDD less sway laterally when walking compared to BAR patients, i.e., patients with RDD have a more stable gait compared to BAR. In the COCE task, we see patients with BAR stabilize their gait and the tachy-reactive Ss become 31.4%, the tachy-reactive persons in RDD decrease, reaching 26.2%. Brady-reactive Ss in RDD are 73.8%, and in BAR – 68.6%.

We perform the same Pearson's cross-tabular analysis for the CCG marker “Steps” to examine the percentage distribution of hypo- and hyper-active participants in the two clinical groups. Pearson's cross-tabular analysis with post-hoc analysis of adjusted residuals shows that in terms of activity there is a significant difference in the percentage distribution between the two clinical groups. The obtained data on the percentage ratio between the hypo- and hyper-active groups in RDD and BAR are presented in Table 3.

**Table 3:** Percentage distribution of hypo- and hyper-active subgroups.

Groups	Hypo-active		Hyper-active	
Task	RDD	BAR	RDD	BAR
**OE**	51.1	63.5	48.9	36.5
**CE**	53.6	61.3	46.4	38.7
**COCE**	44.8	57.7	55.2	42.3

In Table 3, we see that in the OE task 63.5% of patients with BAR have manifest hypo-activity in gait. This means that in 1 min, nearly two-thirds of the examined patients with BAR take a lower number of steps than the average of the norm. In contrast, hypo-activity in RDD patients occurred in 51.1% of participants. On the other hand, in the same task hyper-activity in the gait in RDD was manifested in 48.9% of the participants, while this percentage in BAR was only 36.5%. In the CE task 61.3% of patients with BAR have manifest hypo-activity in gait and 53.6% of RDD participants showed hypoactivity. This means that a larger population of participants with BAR took fewer steps than the average norm when compared to patients diagnosed with RDD. In the COCE task, we see that 55.2% of RDD show hyper-activity in their gait. While this percentage at BAR is only 42.3%. In Table 3, we see that in the three psychomotor tasks (OE, CE, COCE), hypo-active psychomotor in gait is predominantly observed in individuals with BAR and a greater percentage of subjects with RDD exhibit hyper-activity in gait.

**Fig. 1:**
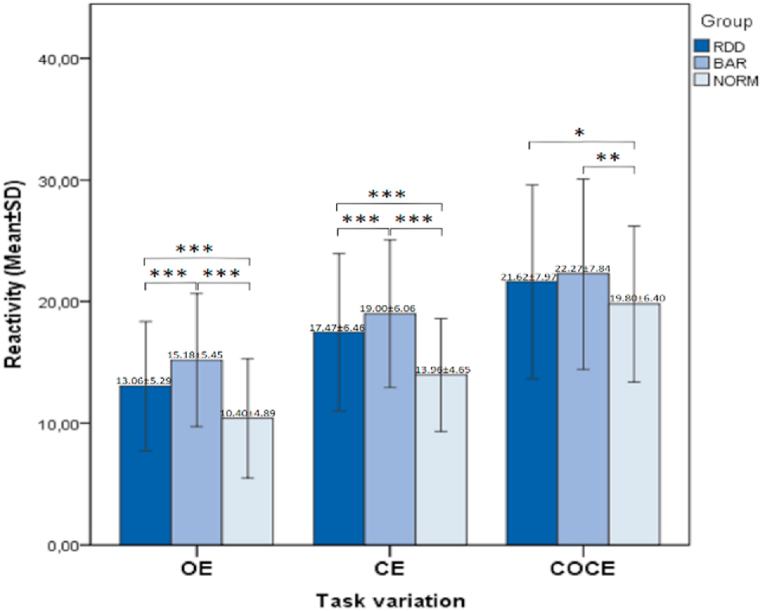
Significant differences in reactivity between groups for the three task variations: OE, CE, COCE. *Legend:* *** – Statistically significant difference - p < 0.005; ** – Statistically significant difference - p < 0.01; * – Statistically significant difference - p < 0.05.

**Fig. 2:**
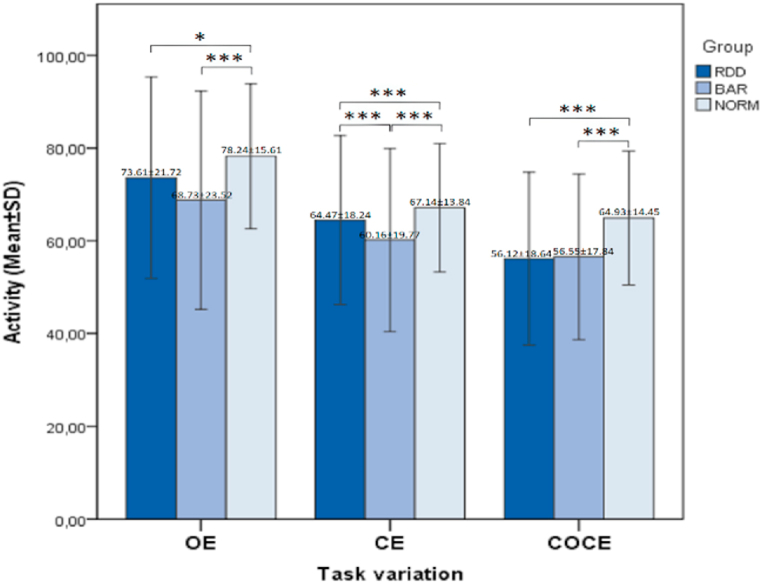
Significant differences in activity between groups for the three task variations: OE, CE, COCE. *Legend:* *** – Statistically significant difference - p < 0.005; ** – Statistically significant difference - p < 0.01; * – Statistically significant difference - p < 0.05.

There is a statistically significant difference between the percentage ratio of hypo- and hyper-active groups in RDD and BAR. The ANOVA analysis of PMA in the three groups (RDD, BAR and NORMS) shows F = 8.067 (p = 0.000) i.e., above the critical value F_crit_ (582, 2) = 3.01 at significance level α = 0.05, which means that there is a statistically significant difference between the distributions in the groups. The Levene's test for homogeneity of variances indicates that the groups do not have equal variances (p = 0.000). From the Games-Howell post-hoc analysis we obtain significant difference between RDD and NORMS (p = 0.024) and a significant difference between BAR and NORMS (p = 0.000). To analyze the significant differences in psychomotor reactivity (PMR) and activity (PMA) between the three groups (RDD, BAR and NORMS) for the three psychomotor tasks (OE, CE and COCE) an ANOVA analysis was performed with post-hoc analysis of the Scheffe. The results show that there are statistically significant differences in PMR between RDD and BAR in the standard Unterberger – CE problem with a value of p = 0.01. There is a significant difference in PMR between RDD and BAR also in the simplified psychomotor task, p = 0.005. The comparison of PMR in NORMS and RDD shows that there is a significant difference between them for all three tasks, as in OE p = 0.005, in CE p = 0.005 and in COCE p = 0.05. There are also significant differences in PMR between NORMS and BAR as in the OE variation the value of p = 0.005; for the task with CE p = 0.005 and for complicated cognitive task p = 0.01. With regard to PMA, significant differences were found only between NORMS and RDD in the three tasks, as in OE p = 0.005, in CE p = 0.01 and in the cognitive task - COCE p = 0.005, as well as between NORMS and BAR in two of the tasks – OE with values of p = 0.05 and for COCE with p = 0.005. Interestingly, no statistically significant difference in PMA was found between RDD and BAR in the three psychomotor tasks. The data from the analyses is displayed on Fig. 1, Fig. 2.

## 4 Discussion

In the descriptive analysis of the data, we found that the three studied groups did not differ significantly according to their demographic and clinical characteristics – gender, age, weight, height, severity of the depressive episode. A large number of individuals with RDD (317), BAR (137) and Norms (182) were studied, with a total mean age 40.7 ± 11.2. Through our study, we show that the objective study of gait in unipolar and bipolar depression makes sense, because between these two groups we can find significant differences in their psychomotor activity and reactivity. In unipolar and bipolar depressions, we can find more significant differences in reactivity than in activity. In bipolar patients, reactivity is significantly slower – brady-reactivity is observed. Brady-reactivity is also present in unipolar patients. It is less pronounced than in bipolar, but higher than in healthy controls (Fig. 1). To a lesser extent, the same trend is found in psychomotor activity. It is most reduced in bipolar patients where hypo-activity is observed. RDD also has a form of hypo-activity compared to the norm, but it is less pronounced than BAR (Fig. 2).

We find that hypoactive individuals with BAR in all three conditions of the Unterberger test (OE, CE, COCE) occupy a higher percentage compared to hypoactive individuals with RDD. Also, with BAR there is a more pronounced form of brady-reactivity. The combination of the mentioned clinical psychomotor disorders – brady-reactivity and hypo-activity can be interpreted as the main sign of inhibited psychomotor. Therefore, from the obtained results (using the CCG method) we can say that depressed patients with BAR are more psychomotor inhibited compared to RDD and Norms, as they have more pronounced brady-reactivity and hypo-activity. Theoretically, on one hand, the significantly more pronounced delay in reactivity and activity in BAR could be explained by the repeatedly established hypodopaminenergy in the striatum [15,33,34] and on the other hand, it could be argued by impairment of cerebral hemodynamics and structural plasticity [35]. These two hypotheses are complementary. Recent studies have shown that dopamine, as well as changes in serotonin and GABA-energetic systems seen in agitated depression, are involved in shaping neuronal plasticity, sensory processing and cognitive functions [19,36]. At the structural level, deficits in GABA and increases in dopamine-energetic systems are associated with alterations in the cerebellum, the meso-cortico-limbic pathway, and the striatum [15,37].

In cases of psychomotor retardation in depression, opposite changes are found – hypodopaminergy in the mesocortical and mesolimbic structures [15,37]. Simultaneous reduction of the thalamus and the sensorimotor network, as well as disturbances in its functionality, are registered. A reduction in the volume of the substantia nigra and the basal ganglia has been described [38]. The indicated differences involved in the mechanisms of agitated and retarded depression support the thesis that their treatment should be diametrically opposed [39]. Some authors suggest that psychomotor agitation in RDD may be a marker for the presence of latent bipolarity. This could explain the resistant response to therapy in some RDD patients and their increased suicidality [31]. The results of our study are comparable to data from previous studies, which found that a large percentage of RDD patients who have depressed mood exhibit objectively measurable hyperactive psychomotor performance. This means that their psychomotor activation is the opposite of their affective disorder. All these data seem to support the idea of Kraepelin, according to whom in depression we can observe polar disorders in the psychomotor, affective and cognitive spheres of development – in the direction of agitation or retardation. Therefore, the point of seeking and finding of psychomotor agitation is in improving the treatment and prevention of more distant negative effects of antidepressant monotherapy. And because the combination of depressed mood and psychomotor activation increases suicide risk [31]. It seems that psychomotor tests are an important objective diagnostic and prognostic marker in psychiatric patients. In gait disorders, we can see that the two groups of depression – unipolar and bipolar, are significantly different from each other in the psychomotor tasks with open eyes and closed eyes. In unipolar individuals, the gait is more stable and agitated compared to bipolar individuals. In the present study we can also see that people with RDD and BAR are psychomotorically more inhibited than the norm. This data is logical and expected, given that the prototype endogenous (melancholic) depressions have inhibited affectivity, thinking and will (melancholic triad) and this inhibition directly affects their psychomotor skills. When studying the dynamics of psychomotor reactivity and activity in the three groups – RDD, BAR and Norms, it turns out that different psychomotor tasks to different extend distinguish the two depressive clinical subgroups, both from each other and from Norms. This differentiation is both for psychomotor activity and reactivity in the three tasks. Unexpectedly, in the case of reactivity, simplified task (OE) is the most sensitive and complicated (COCE) is the least sensitive (Fig. 1). This can be explained by the activation of compensatory mechanisms during the execution of the cognitive task. Similar studies show that in older people over the age of 75, participants are slower in dual cognitive tasks than in single (simple) tasks. The increased complexity of the cognitive task leads to a greater delay in gait. Delay in psychomotor behavior is least pronounced in the simplified task and most pronounced in the complicated cognitive task [40].

The clinical findings are in line with data from previous studies [26,30,39,41]. The results of current analyses show that the overall effect of cognitive tasks of varying complexity is noticeable mainly in gait speed. It turns out that in healthy participants there is a strong relationship between age, cognitive state and decrease in gait speed in conditions of dual cognitive task [42]. Our study shows that the three study groups (RDD, BAR and Norms) did not differ significantly in gender, age, weight or height. There are significant differences in psychomotor activity in the three psychomotor tasks in the three groups. The complicated task (COCE) is the most sensitive to activity disorders (Fig. 2). Next in sensitivity is the simplified version of the task (OE) and third – the standard task (CE). This is a completely new finding in clinical practice and gives significant meaning in the application of the three types of tasks when working with CCG.

### 4.1 Study strengths and limitations

The main strength of this study is that for the first time it is established with such a large clinical sample that the two types of depression are psychomotorically different, both in terms of psychomotor reactivity and activity. Bipolar patients are significantly more psychomotor inhibited than unipolar patients. Both clinical samples more inhibited than the norm. For the first time, the sensitivity of individual equilibriometric tasks to psychomotor disorders in unipolar and bipolar patients is studied. It shows that the most sensitive test in terms of psychomotor activity is the complicated task (COCE), which is logical on the basis of data from the literature, but next in sensitivity is not the standard task (CE), but the simplified (OE). In terms of psychomotor reactivity, the most sensitive task is the simplified one and the least sensitive – the complicated task.

The study also has some limitations: The CCG device is stationary and is used only in laboratory conditions. The measuring perimeter of the machine is limited, which sometimes leads to invalid tests. The device measures a limited number of psychomotor parameters. There is still no clear formula for precise classification of unipolar and bipolar patients based on the CCG parameters Lateral sway and Steps. In our study we do not examine the correlation between type of PD and depressive feelings of guilt or existential emptiness. We did not track the level of anxiety or the type of attachment disorder in depression and their correlation with psychomotor disturbances. After the first CCG examination in depressed patients who were admitted without taking medication, we did not follow the effect of their subsequent prescribed medication. No additional methods are used to examine the severity of the depressive episode. In the examination for the diagnosis of the patients, we rely on an expert assessment by a psychiatrist and a check for the presence or absence of cranio-corpo-graphic pathology. All these limitations of our study are an opportunity for further research and creation of new articles on working with CCGs in psychiatric patients.

## 5 Conclusion

We can hypothesize that RDD and BAR have different neurobiological and psychological mechanisms. This is manifested by the significant differences in their psychomotor skills – their psychomotor activity and reactivity in gait disorders, measured by the CCG. The objective research method itself proves to be rapid, easily replicable, and economical to use when we are working with psychiatric patients. It can be used as a trusting objective relationship between the patient and the therapist to monitor his therapy or objectify his disorders. It seems many objective technologies are used around the world to study psychomotor in differentiating difficult-to-distinguish psychiatric conditions and to monitor the effect of their therapy. Given the data found, we could assume that there is a tendency to use other modern technologies in the study of psychomotor disorders in psychiatric patients.

## Funding statement

This research did not receive any specific grant from funding agencies in the public, commercial, or not-for-profit sectors.

## Author contribution statement

Diana Bogdanova: Conceived and designed the experiments; Performed the experiments; Analyzed and interpreted the data; Contributed reagents, materials, analysis tools or data; Wrote the paper.

## Data availability statement

The authors do not have permission to share data.

## Declaration of competing interest

The authors declare that they have no known competing financial interests or personal relationships that could have appeared to influence the work reported in this paper.
